# Genome Sequence of *Dehalobacter* UNSWDHB, a Chloroform-Dechlorinating Bacterium

**DOI:** 10.1128/genomeA.00720-13

**Published:** 2013-09-19

**Authors:** Nandan P. Deshpande, Yie K. Wong, Mike Manefield, Marc R. Wilkins, Matthew Lee

**Affiliations:** School of Biotechnology and Biomolecular Science, University of New South Wales, Sydney, Australiaa; Systems Biology Initiative, School of Biotechnology and Biomolecular Sciences, University of New South Wales, Sydney, New South Wales, Australiab; Centre for Marine Bioinnovation, University of New South Wales, Sydney, Australiac

## Abstract

The chloroform-respiring bacterium *Dehalobacter* UNSWDHB was isolated from subsurface soil contaminated with a mixture of organohalides, including chloroform. Here, we present its 3.2-Mb genome**.**

## GENOME ANNOUNCEMENT

Trichloromethane, commonly known as chloroform (CF), is a toxic and recalcitrant organohalide with an aqueous half-life of 3,100 years (1), and it is currently ranked 11th on the U.S. Environmental Protection Agency (EPA) priority list of hazardous substances (see http://www.atsdr.cdc.gov/substances/toxsubstance.asp?toxid=16). CF was formerly used as an anesthetic and a precursor chemical in the manufacture of chlorofluorocarbons used as refrigerant gases. For these reasons, CF has been manufactured in vast quantities; for example, from 1983 to 1993, the production of CF averaged 203 million kg per annum in the United States alone (http://www.atsdr.cdc.gov/substances/toxsubstance.asp?toxid=16). Large-scale production and use of CF have led to large-scale pollution due to poor handling and storage practices. Currently, CF exists in 474 of the 1,287 priority polluted sites in the United States (http://toxmap.nlm.nih.gov/). Additionally, CF is a strong inhibitor of microbial metabolic processes, including organohalide respiration (2, 3). This property of CF means that microbial remediation of organohalide-polluted sites is problematic where CF is part of the organohalide mixture.

In recent years, there have been two reports of microbial populations that are capable of using CF as a terminal electron acceptor. The first, in 2010, showed the transformation of CF to dichloromethane (DCM) by a mixed *Dehalobacter* population (4), and the second (from our laboratory) showed CF respiration to DCM; DCM was then transformed to acetate and hydrogen also by a mixed community containing *Dehalobacter* (5). Here, we report the genome sequence of the CF-respiring *Dehalobacter* sp. strain UNSWDHB, isolated from the mixed community completely degrading CF (5).

The UNSWDHB strain was sequenced using an Illumina MiSeq sequencer. A total of 3,787,713 paired-end reads were generated with a read length of 251 bases. This represents the equivalent of 230× coverage. Low-quality bases were trimmed from sequence reads before assembly using the SolexaQA software package (6). *De novo* assembly of the reads was carried out using Velvet 1.2.06 (7) and ABySS 1.3.4 (8) and was tested over a range of *k*-mers, from 51 to 99 bases. The draft assembly generated using ABySS with a *k*-mer value of 63 was found to be optimal when evaluated with parameters, such as cohesiveness (number of contigs, 220), N_50_ (38,707 bp), and the genome size (3,209,125 bp). The draft genome sequence of UNSWDHB has a G+C content of 44.9%.

Initially, the genome of UNSWDHB was compared with that of *Dehalobacter* sp. CF (9), the only other known CF-respiring bacteria. The comparison showed that 246 genes are specific to the UNSWDHB strain, whereas 226 genes are present in the *Dehalobacter* sp. CF strain but absent in UNSWDHB. In a more comprehensive analysis, UNSWDHB was compared with all sequenced *Dehalobacter* strains (9–11). This comparison identified 2,243 core genes common to all sequenced *Dehalobacter* strains. Of five genes specific to the UNSWDHB strain, three genes showed phage-related functional annotations. Also identified in the UNSWDHB genome were genes encoding 17 reductive dehalogenases, 14 of which were shared with *Dehalobacter* sp. CF and three that appeared to be unique to UNSWDHB.

### Nucleotide sequence accession numbers.

This whole-genome shotgun project has been deposited at DDBJ/EMBL/GenBank under the accession no. AUUR00000000. The version described in this paper is version AUUR01000000. The NCBI locus ID for this submission is UNSWDHB.
